# Predictive Crime Mapping: Arbitrary Grids or Street Networks?

**DOI:** 10.1007/s10940-016-9321-x

**Published:** 2016-09-09

**Authors:** Gabriel Rosser, Toby Davies, Kate J. Bowers, Shane D. Johnson, Tao Cheng

**Affiliations:** 1 SpaceTimeLab, Department of Civil, Environmental and Geomatic Engineering, University College London, Gower Street, London, WC1E 6BT UK; 2 Department of Security and Crime Science, University College London, 35 Tavistock Square, London, WC1H 9EZ UK

**Keywords:** Crime prediction, Street network, Burglary, Crime mapping

## Abstract

**Objectives:**

Decades of empirical research demonstrate that crime is concentrated at a range of spatial scales, including street segments. Further, the degree of clustering at particular geographic units remains noticeably stable and consistent; a finding that Weisburd (Criminology 53:133–157, 2015) has recently termed the ‘law of crime concentration at places’. Such findings suggest that the future locations of crime should—to some extent at least—be predictable. To date, methods of forecasting where crime is most likely to next occur have focused either on area-level or grid-based predictions. No studies of which we are aware have developed and tested the accuracy of methods for predicting the future risk of crime at the street segment level. This is surprising given that it is at this level of place that many crimes are committed and policing resources are deployed.

**Methods:**

Using data for property crimes for a large UK metropolitan police force area, we introduce and calibrate a network-based version of prospective crime mapping [e.g. Bowers et al. (Br J Criminol 44:641–658, 2004)], and compare its performance against grid-based alternatives. We also examine how measures of predictive accuracy can be translated to the network context, and show how differences in performance between the two cases can be quantified and tested.

**Results:**

Findings demonstrate that the calibrated network-based model substantially outperforms a grid-based alternative in terms of predictive accuracy, with, for example, approximately 20 % more crime identified at a coverage level of 5 %. The improvement in accuracy is highly statistically significant at all coverage levels tested (from 1 to 10 %).

**Conclusions:**

This study suggests that, for property crime at least, network-based methods of crime forecasting are likely to outperform grid-based alternatives, and hence should be used in operational policing. More sophisticated variations of the model tested are possible and should be developed and tested in future research.

## Introduction

Decades of empirical research demonstrate that crime is concentrated at a range of spatial scales, from neighbourhoods, to census blocks, to street segments, to street corners to individual addresses. In characterising typical patterns, Clarke and Eck (2005) invoke the 80:20 rule (also known as the Pareto principle), which states that 80 % of a problem (here crime) is accounted for by approximately 20 % of potential targets (here places). Examples from the crime and place literature that are consistent with (or more extreme than) this include Sherman et al.’s (1989) finding that in Minneapolis (USA) 50 % of calls for service originated from 3.3 % of the cities addresses or intersections, Budd’s (2001) finding that 1 % of UK households experienced 42 % of residential burglaries, and Bowers’ (2014) finding that 80 % of thefts in bars in London (UK) occurred in 20 % of facilities. Similar findings have been observed at the street segment level: Braga et al. (2011) show that 50 % of street robberies in Boston (USA) occurred on 1 % of segments, while Andresen and Malleson (2011) report that 50 % of vehicle thefts occur on 5 % of segments in Vancouver (Canada). These examples demonstrate that crime is consistently found to be highly concentrated across a range of cities and crime types, with this being observed to be the case at various spatial scales.

Formalising this principle, Weisburd (2015) has recently proposed the *law of crime concentration at places*, which states that “for a defined measure of crime at a specific microgeographic unit, the concentration of crime will fall within a narrow bandwidth of percentages for a defined cumulative proportion of crime”. This is supported by a multiple-city analysis, in which it is shown that between 0.4 and 1.6 % of street segments accounted for 25 % of criminal incidents in data for a number of cities in the US and Israel. Furthermore, this finding is found to remain stable over time, despite significant volatility in absolute levels of crime. This apparent universality has significant implications for analytical approaches which seek to leverage the clustering of crime.

That crime is concentrated, and that the extent of this tends to be stable, has informed criminological theory and, equally importantly, crime prevention practice. Areas in which crime is concentrated represent natural targets for crime prevention effort, since it is in those locations that the greatest impact is likely to be had. A growing number of studies have demonstrated that focusing limited crime reduction resources at high-risk locations reduces crime, while not appearing to displace offending activity elsewhere (Bowers et al. 2011; Braga et al. 2014).

At the individual address level, the Kirkholt burglary prevention project provides a powerful example of this (Forrester et al. 1988). A key component of that project focused on preventing the repeated victimisation of vulnerable homes through improvements to the physical security of recently-victimised households. Following intervention, burglary reduced by more than 50 % across the treatment area, and there was no evidence of spatial displacement to nearby locations. A recent systematic review suggests that such strategies are generally effective at reducing residential and commercial burglary, but not sexual assault (see Grove et al. 2012).

Adjusting the spatial scale slightly, hotspots policing strategies involve the deployment of police resources to high-risk places (e.g. homes, stores, street corners, or subway stations) or hotspot locations comprising a geographical area of up to a few city blocks. Interventions at such locations may include increased police patrols, problem oriented policing (Goldstein 1990), or offender-based strategies. Braga et al.’s (2014) systematic review of hotspots policing suggests that these too are effective at reducing crime, and that crime is generally not displaced to nearby areas as a result of such action.

Geographically focused crime reduction strategies thus appear to be effective. Even at those locations that are generally the most risky, however, crime will not occur all the time, meaning that permanently deploying resources (particularly police officers) to them may be inefficient. Similarly, the dynamic nature of crime patterns—and the phenomenon of space-time clustering in particular—undermines static resource allocation strategies. This is a particular concern given that crime reduction resources are limited. Consequently, a natural evolution in the criminology of place research literature concerns the development, comparison and validation of analytic methods that can be used to best predict the future form and location of crime problems. A number of different methods and, indeed, software tools have emerged which aim to fulfil this requirement. In this paper, we focus on one of these—the prospective mapping technique proposed by Bowers et al. (2004)—and introduce a novel network variant of this approach.

In what follows, we briefly review theories of environmental criminology that relate to the spatial concentration of crime. We then consider research that suggests that crime clusters not only in space, but in space and time, and how this informs methods of crime forecasting. We then discuss spatial units of analysis, and consider in particular why street segments—and not the grid cells currently used in predictive mapping systems—represent a particularly meaningful unit of account. Finally, we introduce and test a network-based prediction method, and compare its accuracy to a grid-based alternative.

### Spatial Crime Concentration

Routine activity theory (RAT; Cohen and Felson 1979) considers crime occurrence through the lens of human ecology, and suggests that direct contact predatory crimes occur as a result of human interaction that emerges as a consequence of everyday activity. People’s routine activities largely dictate what they do at particular times of the day and, in turn, where they will be. Considering the population of a city, the everyday activities of individuals lead to the concentration of people at some places at certain times of the day, and the absence of people at others. At some times and locations people will be stationary, while at others they will be passing through. According to the theory, crime occurs when a motivated offender encounters a victim or target they are capable of victimising, in the absence of a capable guardian who might prevent an offence from taking place. Hence, all else equal, burglaries are to be expected when burglars are at locations when occupancy is low (for example), and robberies are to be expected when robbers are at locations that supply sufficient targets but few (or no) capable guardians. It is people’s routine activity patterns that shape the likelihood of such convergences.

Offenders, victims and capable guardians are, of course, all subject to constraints. In their simplest definition, routines shape *when* particular activities are typically completed, but not necessarily *where*. Complementing RAT, crime pattern theory (Brantingham and Brantingham 1993) considers how human mobility patterns and people’s engagement in legitimate (routine) activities lead to the development of *activity spaces*. Familiarity develops within, near to and along the pathways that connect important or regularly-visited locations in the everyday routines of citizens. In the case of offenders, familiarity brings awareness of criminal opportunities, and, while offenders might commit offences anywhere, they cannot offend in locations of which they are not aware. Moreover, prior awareness reduces uncertainty regarding the likely rewards and risks associated with offending, which, according to the rational choice perspective (Clarke and Cornish 1985), offenders seek to maximise and minimise respectively when making event-level offending decisions. Consequently, the theory predicts that offenders will typically commit offences within or near to those locations with which they are most familiar. With respect to crime pattern formation, geographical hotspots of crime are anticipated to emerge where offender awareness spaces overlap and suitable opportunities for crime are in sufficient supply. Empirical evidence—too vast to review here, but including ethnographic studies (e.g. Bennett and Wright 1984; Wiles and Costello 2000), the analysis of calls for service (e.g. Sherman et al. 1989), and the analysis of crimes detected by the police (e.g. Townsley and Sidebottom 2010)—provides clear support for crime pattern theory.

### Space-Time Clustering

While decades of research demonstrate that crime clusters spatially, evidence also suggests that there is a dynamic character to crime patterns. For instance, consider repeat burglary victimisation of the same home. Evidence (e.g. Osborn and Tseloni 1998) suggests that while some homes may avoid victimisation, others will be victimised many times. It is also apparent that the timing of offences is not random. Instead, repeat offences typically occur swiftly (e.g. Polvi et al. 1991), with the time between offences being too short to suggest that sequential offences at the same location can be explained by the fact that some homes represent more suitable opportunities than others (Johnson 2008). Indeed, such findings suggest a mechanism of event dependency (Pease 1998), whereby the risk of victimisation changes after an offence occurs, if only temporarily. The most parsimonious explanation for such dependency is that, after completing a burglary, offenders return to those locations that they perceive to offer rewards that outweigh the associated risks. Compared with homes that have not been targeted previously, returning to such homes may be particularly appealing, in the short-term at least, since more will be known and hence uncertainty minimised for these homes (see Farrell et al. 1995). Doing so is also consistent with optimal foraging strategies (e.g. Johnson 2014), since offenders will reduce the time spent searching for crime opportunities. Over time, however, things may change: victims of crime, residents in a neighbourhood, or the police (for example) may react, making crime more risky, less rewarding or more difficult to commit. Moreover, an offender’s memory will fade, increasing uncertainty about previously-victimised homes. Thus, returning to burgled homes perceived to be suitable targets swiftly represents a rational foraging strategy.

Empirical evidence from offender interviews (Ashton et al. 1998), the analysis of crimes detected by the police (Everson and Pease 2001), and simulation experiments (e.g. Johnson 2008; Short et al. 2008; Pitcher and Johnson 2011) provide clear support for this explanation. In addition, research (e.g. Bernasco 2008; Johnson et al. 2009b; Summers et al. 2010; Bernasco et al. 2015) demonstrates that, as well as returning to already-victimised homes, burglars often target (again swiftly) the neighbours of burgled homes and those nearby for similar reasons (e.g. Johnson and Bowers 2004). Such events have been termed *near repeats* (Morgan 2001) and, in terms of crime pattern formation, empirical evidence conducted using techniques initially developed in the field of epidemiology (e.g. Townsley et al. 2003; Johnson and Bowers 2004; Johnson et al. 2007a) suggests that such targeting behaviour leads not just to spatial clusters of crime, but to *space-time* clusters of crime. Simply put, crime moves. However, this is not to say that crime hotspots are temporary, but that within an area—be it a high crime area or not—there is an evident regularity to precisely where and when crimes take place. In all studies that have so far examined such patterns (for an international comparison, see Johnson et al. 2007a; and for a review, see Johnson and Bowers 2014), it appears to be the case that when a crime occurs at one location, others are temporarily more likely nearby.

### Prospective Mapping

Traditional methods of crime mapping, such as kernel density estimation (KDE), are used to generate risk surfaces that indicate where crime has previously clustered. As such, they consider the location of crime events but ignore their timing. Inspired by the above findings, Bowers et al. (2004) proposed a method of predictive crime mapping, named ProMap, that models the way in which crime clusters (or appears to spread) in space *and* time. To do this, the expected risk at a location for a particular period (usually the next day, few days or the next week) is estimated as a function of the density of crime that has occurred at or near to that location. However, events are also inversely weighted according to when they occurred, so that more recent crimes receive a greater weighting. The simplest form of the function sums the product of inverse time and distance weights given to each crime in the data set for the locations of interest (e.g. a series of grid cells).

In a series of studies (Bowers et al. 2004; Johnson et al. 2007b, 2009a) that have examined residential burglary, prospective mapping (‘ProMap’) has been shown to offer a modest but reliable predictive gain relative to KDE maps, particularly where the ProMap models used have incorporated data on housing density and the location of major roads. In an attempt to establish the usefulness of this technique in practice, a field trial of ProMap was undertaken in collaboration with the East Midlands Police (UK) and the findings disseminated via a Home Office research report (Johnson et al. 2007b). The application developed as part of that research identified and displayed high-risk grid squares (100 m by 100 m) against a backdrop of the street network to clearly delineate the areas of suggested intervention. Consultation with police practitioners suggested that they thought the maps were useful in an operational context. However, as is true with so many crime reduction interventions (Knutsson and Clarke 2006), problems were experienced with implementation on the ground. Due to organisational changes, resources were not devoted to using the maps in practice as much as had been anticipated prior to the study, so unfortunately it was difficult to evaluate the potential of the system in terms of real-world crime reduction.

Subsequent to the East Midlands trial, approaches based on the ProMap approach have been implemented in other areas of the UK. In Greater Manchester (UK), for example, Fielding and Jones (2012) developed their own system which followed the same principles as those described above. Over an initial 12-month implementation interval, intervention included increased guardianship provided by police patrols and other emergency service staff, and burglary was found to decline by 27 % in the treatment area. This occurred in the context of a force-wide increase of 7 %, and a reduction in burglary of 10 % in the next most similar area. Other trials have been implemented and a common feature of them is that the unit of analysis—in terms of the mapped regions of risk—is grid-based, often with the areas of risk displayed being 50 m by 50 m or larger.

### Other Predictive Approaches

A closely related approach was proposed by Mohler et al. (2011), who used a self-exciting point process (SEPP) model to generate predictions of future crime risk. The distinction between the two methods (ProMap and SEPP) is essentially technical, since both methods are motivated by the literature on repeat and near repeat victimisation discussed above. SEPP models, typically used to model disease contagion or earthquake aftershocks, however, model both how the risk of an event diffuses in space and time, and how enduring characteristics of a location might influence the likelihood of future events. Another advance in Mohler *et al.’s* study was the use of maximum likelihood methods to estimate model parameters. Relative to the simplest ProMap model described above, this SEPP model was found to offer superior predictive accuracy. Mohler et al. 2011’s method has subsequently been developed commercially (as *PredPol*) and is used in a number of policing departments in the USA and UK. Like the systems described above, it identifies grid cells that are expected to be at the greatest risk in the near future, which can be plotted against the backdrop of an area map.

Risk terrain modelling (RTM) takes a different approach to the prediction of risk, focusing not on the crime distribution itself, but on the estimation of how conducive locations are to crime (Kennedy et al. 2011). Hence, key aspects of the environment that are considered criminogenic—such as bars, schools and bus stops—are layered together in a geographical information system. To produce an overall risk surface, individual features are weighted and an additive model used to combine the risks associated with each layer. Again, risk maps are presented by the demarcation of areas of risk using a grid of uniformly sized cells. As well as focusing on environmental factors to generate predictions, a further distinction of this method is that the forecast horizon for which predictions are made (e.g. the next 6 months) is typically much longer than for either ProMap or PredPol, for which predictions are for much shorter intervals, such as the next day.

Our discussion of predictive methods is necessarily brief and serves only to motivate what follows. Other approaches exist, and the interested reader is referred to the work of Gorr and Olligschlaeger (2002), who use time series approaches to generate area-level predictions; Olligschlaeger (1997), who uses artificial neural networks; Rey et al. (2012), who use a conditional spatial Markov chain technique; and Cheng and Adepeju (2013), who examine the accuracy of SatScan at predicting future crime locations. These methods each have a slightly different focus and predict crime at different spatial scales, but all do so for area boundaries or grid cells. Readers might also consult review articles such as those produced by Groff and La Vigne (2002) and Bowers and Johnson (2014).

### Moving to a Street Network-Based Method

Research on predictive crime mapping has thus increased in the last decade, but it is evident that the overwhelming majority of efforts have involved the generation of predictions using areal units such as two-dimensional grid cells or administrative regions. While these have shown promising results, however, a number of issues suggest that alternative spatial units—and the street network in particular—may be more meaningful for analysis of this type.

The utility of studying crime at the street level is illustrated by an increasing body of research (see Weisburd et al. 2012 and below), and there are a number of reasons why network-based models are appropriate for the description and prediction of crime concentrations. The first is simply that much urban crime and policing activity happens on (and along) streets, so that they represent a more meaningful representation of location than arbitrarily-defined grid squares. In addition, the use of street segments is well-aligned with the movement towards the use of micro-units within criminology (Brantingham et al. 2009). There is growing evidence that crime variability can best be explained at fine spatial scales: Steenbeek and Weisburd (2016), for example, recently showed that 58–69 % of the variability of crime can be attributed to street segments. This also highlights the utility of network-level analysis in guarding against the ecological fallacy (Robinson 1950). This refers to the assumption that risk will be uniform across an area, which may be particularly problematic in cases where street segments that are co-located within an area (or grid cell) experience very different risks. This highlights a potential shortcoming of grid-based methods, which may fail to identify a high-risk segment if it is ‘cancelled out’ by a low risk segment in the same grid cell: this has clear implications for predictive accuracy and effective police deployment.

The issues outlined above are amplified further by the fact that research demonstrates that *features* of the street network may themselves influence levels of crime. In particular, studies have shown an association between the likelihood that people (offenders) will be aware of particular street segments—and the criminal opportunities they provide—and crime risk: a finding predicted by crime pattern theory. For example, using police data for West Yorkshire (UK), Armitage (2007) shows that homes located on cul-de-sacs were at a lower risk of burglary than those located on through roads, while a similar relationship was found for violent crime during Operation Cul de Sac in Los Angeles (Lasley 1998). Beavon et al. (1994) show that the risk of (various types of) crime on street segments in Ridge Meadows (Canada) was positively associated with the number of roads to which a street segment was connected (a simple index of permeability). Using data for Merseyside (UK) and simple graphic theoretic metrics, Johnson and Bowers (2010) show that burglary risk was associated with street type and the (number of different) types of streets to which a street segment was directly connected; the more major roads a segment was connected to, the greater the risk. Davies and Johnson (2014) use a more thorough approach to analysis, using the graph theory metric *betweenness* to estimate the likely movement of people through (and hence their awareness of) street segments. In line with previous studies, all else equal, they find a positive association between estimated street segment usage and burglary risk for the city of Birmingham (UK). Using a similar approach, Summers and Johnson (2016) find the same pattern for incidents of outdoor serious violence in London.

These findings suggest the importance of the network in understanding the overall (long-term) risk on segments, but there is also reason to expect that it will play a role in short-term dynamics. Returning to our discussion of offender targeting strategies, if burglars revictimise homes and their neighbours due to the awareness they develop of these homes, it seems reasonable to suggest that risk might spread not just to neighbours, but also to locations on directly connected street segments (Johnson and Bowers 2007; Davies and Bishop 2013; Johnson and Bowers 2014). These segments are likely to also lie in the awareness spaces of the offenders in question, who may also gain increased awareness during the process of burgling homes. In grid based approaches, this spread of risk is typically manifested as a process which acts uniformly in all directions. This is, however, unrealistic, since crime is more likely to spread in some directions than others: some areas are well-connected, whereas others may be separated by barriers such as rivers, railway tracks and so on. Most fundamentally, it is the street network that determines what it is for two places to be ‘near’: they may be close as-the-crow-flies, but far in terms of true travel distance. The street network encodes the connections between segments, and hence determines the physical pathways along which offenders can travel (and develop awareness) and along which risk can spread (Johnson and Bowers 2007; Davies and Bishop 2013).

The use of the street network also has practical motivation. In general, grid squares—which could potentially intersect gardens, physical features (e.g. lakes) or barriers (e.g. railway tracks)—are less well-defined targets for operational deployment than particular street segments. Similarly, a grid cell might easily contain one or more unconnected street segments, making patrol plans ambiguous and difficult to follow. Surprisingly little has been written on the topic of map usability (see Bowers and Johnson 2014 for an account of this), but it seems sensible to assume that intended routes that are physically viable and integrated (Bowers et al. 2004) would be preferred to fragmented alternatives. Presumably, when faced with a map that defines areas rather than streets, patrolling officers stick to the accessible routes within the square, making large areas of the map redundant.

The principal aim of the current study is to examine the potential of one network-based crime prediction method. Here, we focus on a network-based variant of ProMap, though we emphasise that the overall approach could be applied to other methods. Since ProMap is essentially a kernel-based method, its adaptation to the network setting relies on the translation of kernel density estimation to network space, for which we build on the approach developed by Okabe et al. (2009). This method has not previously been applied to dynamic prospective crime mapping, and we further extend the approach here by adding a temporal element. The resulting method therefore mirrors the prediction strategy introduced in ProMap, but with calculations based on network space.

To the authors’ knowledge, there is only one (Shiode and Shiode 2014) other attempt to produce a prospective network-based crime mapping system. This aimed to detect emerging crime concentrations at the street level by performing repeated sweeps of the network using a flexible search window as new incidents emerge. The result of this geosurveillance method is an early warning system which generates micro-level ‘alarms’ (from the level of an individual street address upwards). This differs from the current endeavour in two ways. The first is that the geosurveillance method relies purely on patterns in historic data to make predictions, whereas the current method propagates risk down the network in accordance with theories of offender behaviour (the model proposed by Davies and Bishop (2013) also does this, but has not been applied to real data). The second is that the geosurveillance method has not been tested for predictive accuracy; in other words, its capacity to identify the locations of future crimes is not known. The analysis presented here is therefore the first attempt to examine the predictive accuracy of a prospective network-based mapping system. To avoid confounding the processes modelled and the units of analysis employed, we compare the performance of a grid- and network-based variant of ProMap, both of which are calibrated using a maximum likelihood method.

## Methods

In this section, we outline details of our predictive approach and describe the data analysed. Although the overall predictive approach is conceptually similar to previous approaches, the network context introduces a number of technical challenges; we will describe these and the way in which they are overcome. We also introduce the protocol used for the fitting of parameters and methods used to measure the predictive performance of the algorithm.

The setting for the present study is a large city in the UK. The socio-demographic characteristics of the area are broadly in line with those of other major UK cities, and there are no distinctive geographical features which we would expect to influence the distribution of crime substantially.

### The Street Network: Data and Representation

Throughout the network-based analyses, space is represented in terms of the street network, so it is logical to begin by describing how it is structured. Here, we use the Integrated Transport Network data provided by Ordnance Survey (OS) via its MasterMap product. This includes both physical and contextual information.

To perform calculations on the network, it is necessary to represent it in formal mathematical terms. This can be done using terminology from graph theory (see Bollobás 2002), which concerns the structure of pairwise relationships between discrete entities. Formally, a *network* $G=(V,E)$ is a set of *vertices*, *V*, together with a set of *edges*, *E*, which connect pairs of vertices. In basic terms, a network is simply a collection of objects, some pairs of which are linked: vertices that are connected by an edge are said to be *adjacent*. The *degree* of a vertex is defined as the number of other vertices to which it is adjacent.

**Fig. 1:**
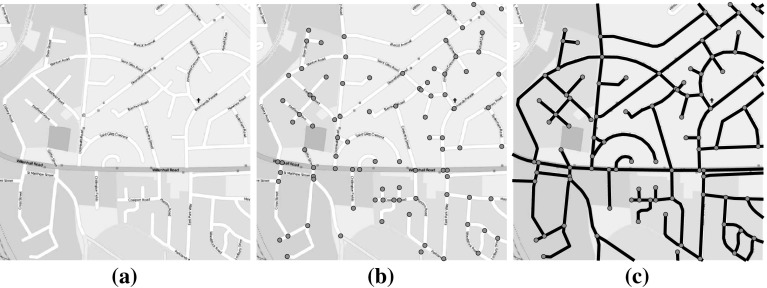
The construction of the primal representation of a street network: **a** the original map, **b** nodes placed at each junction, **c** links added between any pair of junctions connected by a street segment

The most natural way to express a street network in this form is to take vertices to represent junctions (i.e. points at which roads intersect) and to place an edge between any pair of junctions that are directly connected by a street. Edges therefore represent street segments: the sections of road between immediately-neighbouring junctions. This is referred to as the ‘primal’ representation (Porta et al. 2006b), and an example of its construction is shown in Fig.  1. Although other representations exist (Jiang and Claramunt 2004; Porta et al. 2006a), the primal approach is taken here because it is the only one that preserves geo-spatial information: metric distance, for example, is not well-defined in other representations. In the form used here, each edge has an associated length, and points on the network can be identified uniquely by specifying how far along a given edge they lie.

### Police Recorded Crime Data

The prediction and evaluation presented here are based on police recorded crime data for a major city in the UK. These crime records comprise all incidents of residential burglary that occurred between 1st July 2013 and 31st August 2014, inclusive, of which there are 5,862. For each incident, the location is provided in terms of British National Grid coordinates, with a resolution of 1 metre. Temporal information is provided in three forms: the report date, and a ‘start’ and ‘end’ time for the estimated window during which the crime could have occurred. In our analysis, we use the start of the window—the earliest point that an incident could feasibly be prevented—as a proxy for the true event time. A more sophisticated approach to dealing with temporal uncertainty using aoristic analysis has previously been presented by Ashby and Bowers (2013), but we do not consider that here to avoid introducing additional complications to the pre-existing technical challenges. We did, however, repeat the analyses described below using the end of the window, with our results indicating that the optimal bandwidths and predictive accuracy results for the two cases are statistically indistinguishable (data not shown). We conclude, therefore, that in the case of the current dataset, the choice of temporal data has no significant effect on the prediction process; this may, of course, differ for other regions and crime types.

**Fig. 2:**
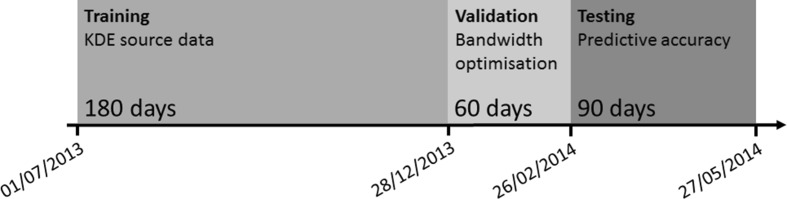
Overview of the methodology in this study, showing the three divisions created from the crime data

As we describe in detail below, it is necessary to divide the data into three non-intersecting sets, which is common practice in statistical applications (Hastie et al. 2009). This is illustrated in Fig. 2. The first 180 days of the data are used to ‘train’ the prediction model. The next 60  days are used to find optimal prediction model parameters and are termed the validation set. Finally, the remaining 90 days are used to assess the predictive accuracy of the model and are denoted the testing set.

Since one of our predictive methods is network-based, it is necessary to express the location of each incident in terms of its position on the street network. We therefore ‘snap’ each incident to the closest street segment. We verified that the Euclidean distance involved in projecting each point was less than 50 m for all incidents considered here; in fact, the mean distance was 18 m. This snapping process transforms the location of each incident to a network point, specified in terms of its distance along the segment on which it lies. Together with the temporal data, these locations form the data for our analysis. Because the distances involved are so small, and because the process is applied for the data used to both calibrate and test the predictive models presented below, we do not believe this introduces significant bias to our analysis.

### Grid and Planar Predictive Methods

The new predictive method described here is conceptually similar to the original grid-based ProMap algorithm developed by Bowers et al. (2004). For a target time *t* and location *s*, the prospective risk level at a given location (a relative measure) is calculated by summing risk contributions from all preceding crimes that are sufficiently close in space:

1 $$\begin{aligned} \lambda _{\mathrm {grid}}(t, s) = \sum _{0 < c_i \le \tau ; e_i \le \nu } \left( \frac{1}{c_i}\right) \left( \frac{1}{1+e_i}\right) \end{aligned}$$

where $c_i$ is the number of weeks that have elapsed since crime *i*, $e_i$ is the number of 50 metre grid cells between location *s* and the location of crime *i*, $\tau$ is the maximum temporal lag (the temporal bandwidth) and $\nu$ is the maximum spatial lag (the spatial bandwidth). The crimes included in the summation are denoted *source* crimes; each crime is represented as a point in time and space, $(t_i, s_i)$.

We now generalise this original approach to a continuous two-dimensional planar space, so that none of the variables are expressed in terms of discrete units such as grid cells or weeks. This is an important step towards formalising the approach mathematically and will make comparisons with the network model (detailed below) more straightforward. We also modify the form of equation (1), so that the function within the sum is normalised (i.e. its integral over all time and space is 1), again to ensure that the comparison is fair. Both of these processes are relatively minor: the modified form remains fundamentally similar to the original ProMap. By summing over a continuous, normalised function, however, the resulting method becomes a form of space-time kernel density estimation (STKDE).

Kernel density estimation is a statistical technique which allows a continuous probability distribution to be estimated from empirical data. It is based on the concept of a *kernel* function, which is a continuous normalised functional form that is peaked (or ‘centred’) at a defined point. To produce an estimate from a set of data points, one such kernel function is centred at each data point, and the sum of these is taken. This essentially produces a ‘smoothed’ version of the data, in which each point is replaced by a ‘bump’ and these are superimposed upon each other. For spatial data, this produces a surface in which the influence of each point is distributed across its immediate vicinity, while for temporal data the influence is spread through time. In the context of crime prediction, these effects can be taken to reflect spatio-temporal propagation of risk, and it is clear that the ProMap approach is an informal version of this principle.

As for ProMap, we assume that the temporal and spatial components of the kernel function are separable. This common assumption is a reasonable simplification in the absence of any evidence to suggest that the function should be more complex. We also make the same assumption that is implicit in ProMap, namely that the spatial propagation of crime risk is isotropic, i.e. it extends uniformly in all directions. This is a necessary approximation as the kernel function is global and therefore represents the spread of risk at all source target times and locations. Variants of the KDE have been developed to relax this isotropic assumption, such as the variable bandwidth nearest neighbour approach (Mohler et al. 2011), but these are not without complications, and are beyond the scope of this study. With these two assumptions in mind, we define two kernel components, $f(\Delta s)$ and $g(\Delta t)$, which represent the spread of risk from a single source to a target over a time lag of $\Delta t$ and a straight-line distance of $\Delta s$, respectively. For any particular source *i*, these source-target distances are given by $\Delta s = \Vert s - s_i\Vert$ (where this notation denotes the Euclidean distance between *s* and $s_i$) and $\Delta t = t - t_i$, and the planar STKDE is then given by

2 $$\begin{aligned} \lambda _{\mathrm {planar}}(t,s) = \sum _{i: t_i<t} f(\Vert s - s_i\Vert ) g(t - t_i). \end{aligned}$$

For our particular form, we choose an exponentially decaying function of time and a linearly decaying function of distance, as follows:

3 $$\begin{aligned} f(\Delta s)&= {\left\{ \begin{array}{ll} \frac{h_S - \Delta s}{h_S^2} &{}\hbox { if }\Delta s \le h_S \\ 0 &{}\hbox {otherwise} \end{array}\right. } \end{aligned}$$

4 $$\begin{aligned} g(\Delta t)&= \frac{1}{h_T} \exp \left( -\frac{\Delta t}{h_T}\right) , \end{aligned}$$

where the parameters $h_T$ and $h_S$ are the temporal and spatial bandwidths, respectively, which must be defined (see below). Our choice of temporal kernel is motivated by previous research concerning the time course of repeat victimisation (e.g. Townsley et al. 2000; Sagovsky and Johnson 2007) which suggests that the distribution of inter-event times is approximately exponential. In the spatial case, however, little evidence exists concerning the form of propagation, and so the linear kernel was chosen as the simplest possible form. Both of these functions are simple to implement and commonly used for KDEs (Genton 2002); however, we make no claim about the optimality of the forms used, and our aim in this respect is only to explore the capability of the overall approach. Our method could easily be extended to use any other valid kernels, of which several possibilities exist. Nevertheless, one feature of the linear kernel does have an important advantage for our current purposes: the fact that it is zero-valued at distances greater than $h_S$ naturally imposes an upper threshold on the distance $\Delta s$ over which risk may be transmitted. This is especially important when dealing with network-based predictions, as we shall show.

### Network-Based Kernel Calculation

**Fig. 3:**
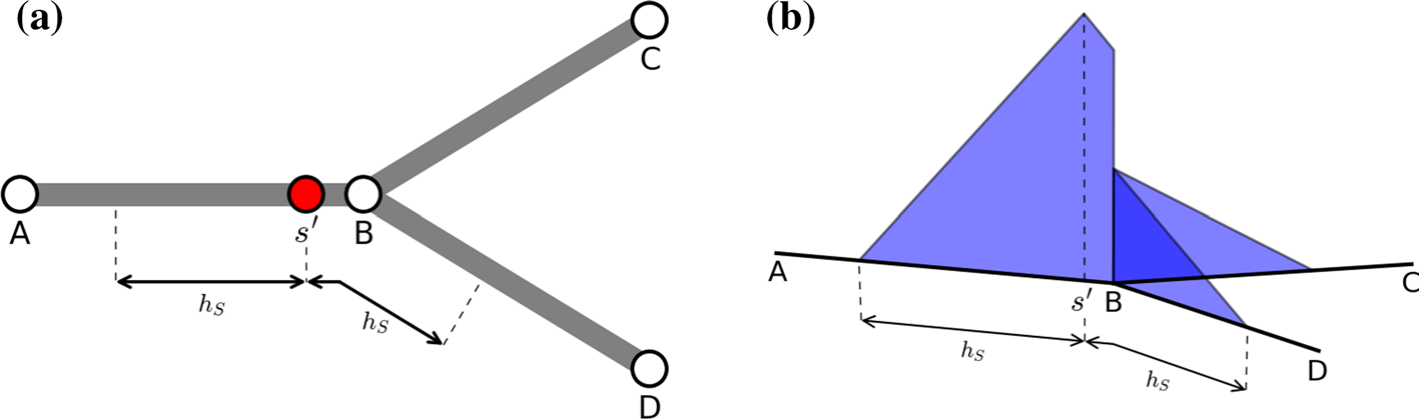
Kernel calculation on networks: **a** for a kernel centred at $s'$, a one-dimensional kernel function must be adapted to apply to each of the branches *BC* and *BD*; **b** the ‘equal-split’ approach, in which the remaining density at a junction is divided equally between the ongoing branches (this shows a linear kernel as used in our work)

The task of kernel density estimation is more complex for networks than it is for classical Euclidean spaces. Network space is fundamentally one-dimensional, since streets are linear, however at nodes (road junctions) the one-dimensional line ‘splits’ (see Fig. 3a) and the domain has a tree-like structure. Furthermore, there are multiple possible routes between two locations on a network; for example, it is possible to circumnavigate a block both clockwise and counter-clockwise. We must therefore redefine our kernel function to take this new spatial representation into account. We ultimately require a solution in which the risk density decays linearly along an edge, so that the network KDE remains comparable with the planar KDE.

We start by changing the notation to show the explicit dependence on the location of the source, $s'$, and the network path that connects *s* with $s'$, denoted *p*. Our spatial network kernel function becomes $k^{(p)}_{s'}(s)$. We apply the method developed by Okabe et al. (2009) for evaluating a KDE on a network, based around the concept of an ‘equal-split’ kernel. In this approach, risk propagates away from the source location $s'$ and, whenever a vertex is reached, is divided equally between all subsequent branches. Figure 3b illustrates this: the value of the kernel at *B* is split equally between *BC* and *BD*. Formally, if $n^{(p)}_1,\ldots ,n^{(p)}_{m(p)}$ denote the degrees of the *m*(*p*) vertices on a path between $s'$ and *s*, and $\Delta s^{(p)}$ denotes the length of the path, then the network kernel is defined as:

5 $$\begin{aligned} k^{(p)}_{s'}(s) = \dfrac{f(\Delta s^{(p)})}{(n^{(p)}_1 - 1)\ldots (n^{(p)}_{m(p)} - 1)} \end{aligned}$$

Using this function as the kernel in a network-based version of the estimator given in (2) can be shown to produce unbiased estimates (Okabe et al. 2009).

Equation 5 is valid for a single path between points *s* and $s'$. In reality, multiple paths may exist between *s* and $s'$ and all must be taken into account when computing the full contribution of the source at the target. In the event of a network cycle existing, which is the case in almost all real street networks, there are in theory an infinite number of ways to travel between two locations in the absence of any maximum distance parameter. For example, one could walk any number of times around any given block en route. Cycles such as these are necessarily ignored. The total contribution of the source at the target is then calculated by summing over all non-cyclic paths between them:

6 $$\begin{aligned} k_{s'}(s) = \sum \limits _{p:s'\rightarrow s} k^{(p)}_{s'}(s). \end{aligned}$$

The temporal kernel component $g(\Delta t)$ remains unchanged and can be combined with Eq. (5) to obtain a *network-time KDE* (NTKDE). Combining Eqs. 2–6, we arrive at the full expression for the network KDE:

7 $$\begin{aligned} \lambda _{\mathrm {net}}(t,s)&= \sum \limits _{i:t_i< t} g(t - t_i) k_{s_i}(s) \nonumber \\&= \sum \limits _{i:t_i< t} \dfrac{1}{h_T} \exp \left( -\dfrac{t - t_i}{h_T}\right) \left( \sum \limits _{\begin{array}{c} p:s_i\rightarrow s \\ \Delta s_i^{(p)} < h_S \end{array} } \dfrac{ h_S - \Delta s^{(p)}_i }{h_S^2(n_1 - 1)\ldots (n_{m(p)} - 1)} \right) . \end{aligned}$$

In the final summation of Eq. (7), we have included the explicit condition that the path length, $\Delta s_i^{(p)}$, should be less than the spatial bandwidth $h_S$. This is an important aspect of the efficient implementation of this algorithm, which essentially proceeds by carrying out an exhaustive search for all non-cyclic network paths that satisfy this criterion.

### Optimal Bandwidth Selection

The STKDE and NTKDE algorithms described above both require the specification of two bandwidth parameters, $h_S$ and $h_T$. The bandwidths have a major effect on the predictions generated, so it is vital to select appropriate values. Myriad methods exist for this purpose, including selecting values manually based on the desired appearance of the output, using a plugin bandwidth estimator and applying statistical cross-validation techniques (Sheather 2004; Arlot and Celisse 2010).

**Fig. 4:**
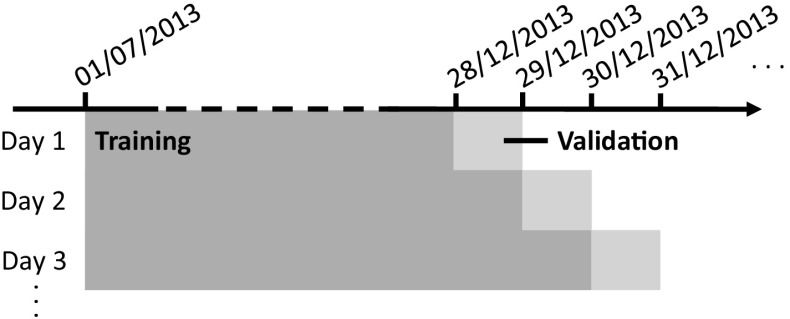
Illustration of the bandwidth optimisation process. For each of 60 days of validation data, all crimes on previous days are used to construct a prediction model, which is evaluated on that single day only

In this study, we employ a method closely related to cross-validation that is appropriate for time series data (Bergmeir and Benítez 2012). The process is summarised in Algorithm 1 and Fig. 4. Broadly speaking, this approach involves optimising the log likelihood over a 60 day set of validation data. On each validation day, the crimes on all preceding days are used to construct an STKDE or NTKDE. The likelihood is then given by the product of the values of the KDE at the times and locations of the validation day crimes. To avoid numerical errors that arise from taking the product of many small numbers, it is better to use the logarithm (log) of the likelihood. One issue that can arise with this approach is in cases where the KDE has a value of zero for one of the validation crimes, which can arise when an incident occurs too far away (in space or time) from all previous crimes. This is problematic because the logarithm of zero—the likelihood value in such cases—is undefined. To avoid this, any log likelihood values below −27.6 ($=\log (10^{-12})$) are manually set to this value, thus ensuring validity in all cases (we do this rather than set the values to be infinite simply to maintain real values). Essentially, this process imposes a minimal level of confidence in any model—albeit a very low one—even if it has absolutely no ability to account for one or more crimes in the validation set. This is purely a technical point, however, since only cases which have little explanatory value are affected.

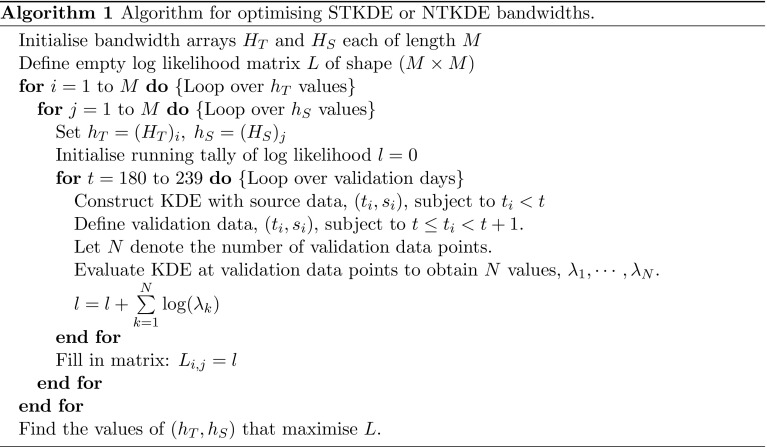



### Assessing Predictive Accuracy

We evaluate the predictive accuracy of the STKDE and NTKDE models using the hit rate (Bowers et al. 2004). In order to define the hit rate, we must first specify the areal unit on which we will make our predictions. Both the STKDE and the NTKDE provide point estimates of the risk density, therefore they can be used to generate predictions on any arbitrary areal unit by defining a number of representative sample points within that unit, evaluating the KDE at those points and taking the mean value. In keeping with other crime prediction methods (Mohler et al. 2011), we use a square grid of side length 150 m as the areal unit for assessing the STKDE. We generate 15 sample points at random within each grid cell and use the mean value as an approximation to the risk level within that cell. In the case of the network-based approach, we use the network segment as the areal unit. In this case, we place sample points approximately every 30 m along each segment, again taking the mean value to represent the risk level. The hit rate metric naturally takes the differing segment lengths into account.

A detailed description of the hit rate calculation is given by Bowers et al. (2004). Briefly, this involves placing all areal units for a given prediction time window in ranked order on the basis of their predicted risk, starting with the highest. For any given level of coverage—calculated as a proportion of the total area in the planar case and total network length in the network case—the highest ranked units are then identified, up to the point where their cumulative coverage is equal to that required. The selected units are then compared with the testing data to see how many (future) crimes are ‘captured’, meaning that they fall within the selected region. The hit rate is expressed as the proportion of crimes captured relative to the total number in the testing data. The behaviour of this hit rate at varying levels of coverage can then be examined. Just as for the optimal bandwidth selection, a prediction time window of 1 day is used and the window is advanced by 1 day per iteration (see Fig. 4 for reference). As illustrated by Fig. 2, we assess the hit rate over 90 predictive iterations (i.e. 90 days). These are either aggregated to obtain a mean hit rate, or considered separately to determine statistical significance (see below).

**Fig. 5:**
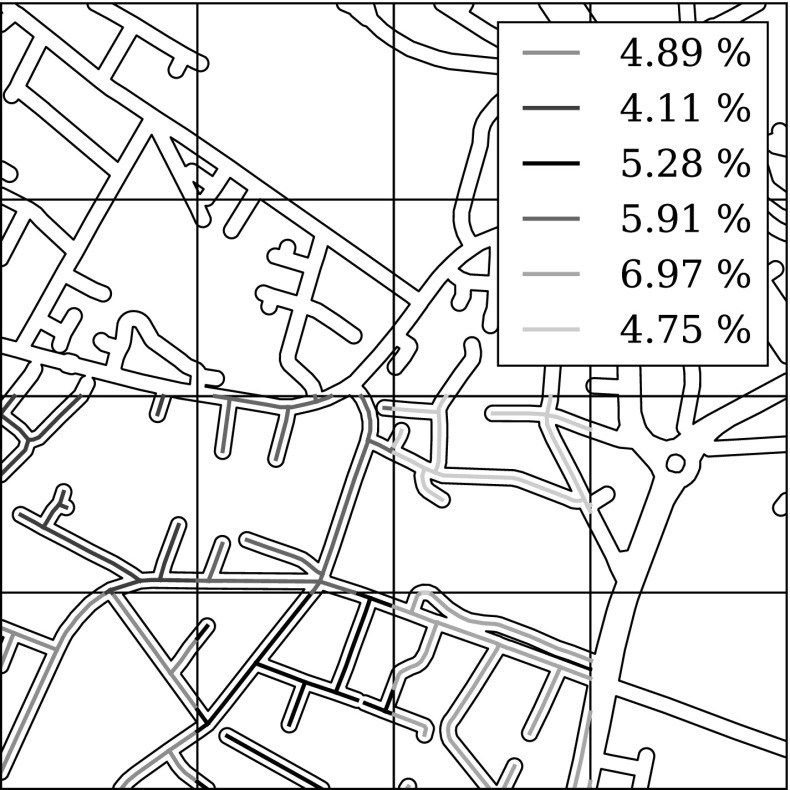
Illustration of the network intersection coverage measure. The plot shows a portion of the network overlaid with grid squares. *Coloured lines* show the intersection between the network and selected grid squares. The values in the legend show the network intersection coverage; for reference, if coverage was measured by area alone, each grid square would account for 6.25 % (Color figure online)

When comparing planar and network-based methods it is important to make sure that the comparison is fair. While network coverage may be linked fairly straightforwardly to the amount of policing resources required (e.g. based on a rough walking speed), the situation is less clear in the case of grid squares. As discussed in the introduction, the number of street segments that lie within a grid cell varies depending upon its location. We cannot therefore directly compare the results by area coverage with those by network coverage. To resolve this difficulty, we define the ‘network intersection coverage’ of a grid-based method as the total network length that intersects with the selected grid squares, divided by the total network length. This measure is illustrated in Fig. 5 and provides a common reference point by effectively translating grid cells into regions of the network. Implicit within this approach is the assumption that police officers would attempt to cover the full extent of the network intersection when targeting a cell. In practice, police officers are more likely to choose one or two representative segments for their attention, reducing the effective coverage. This issue is beyond the scope of the present study, as it would require detailed studies of police patrol habits.

Having computed the predictive accuracy of the two methods, it is possible to compare mean values directly. However, the statistical significance of any such result is unknown. Rather than pooling all 90 daily prediction accuracy measures to compute a mean value, we may consider the values for the two methods as a paired daily time series. Dealing with this representation is complicated by the possibility of temporal autocorrelation in the values, due to the fact that crime levels are themselves correlated in time (Johnson and Bowers 2004). We therefore make the simplifying assumption that the difference in predictive accuracy between two methods is independent of the underlying crime rate, meaning that the time series of the *differences* comprises independent and identically distributed random variables. We verify that this assumption is justified by computing the autocorrelation and showing that it is below the 5 % confidence interval. The two methods may then be compared using standard statistical tests for paired observations, such as Wilcoxon’s signed-rank test (WSR; see Diebold and Mariano 1995). WSR is a distribution-free test that is used to compare two related samples by assessing whether their mean population ranks differ. The test statistic is given by

8 $$\begin{aligned} W = \sum \limits _{i=1}^{90}\left[ R_i \,\mathrm {sgn}(y_{\mathrm {net},i} - y_{\mathrm {grid},i}) \right] , \end{aligned}$$

where $y_{\mathrm {net},i}$ and $y_{\mathrm {grid},i}$ denote the hit rate on day *i* from the NTKDE and STKDE respectively, $R_i$ is the rank of the $i^{\mathrm {th}}$ difference, and sgn is the sign function. By convention, $\mathrm {sgn}(0) = 0$, meaning that exact matches are not included. The statistical significance of *W* can be obtained using a lookup table. We use a single tailed lookup to assess whether one method is significantly better than the other.

## Results

### Optimal Bandwidth Selection

**Fig. 6:**
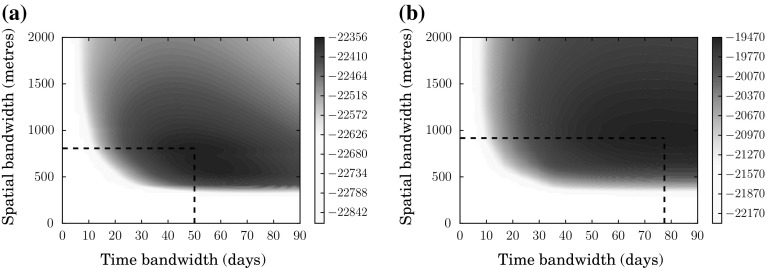
Likelihood surfaces used to compute the optimal temporal and spatial bandwidth in the STKDE (**a**) and NTKDE (**b**). Likelihood values below the 25th percentile are not shaded for clarity. *Dashed lines* indicate the location of the maximum

Figure 6 shows the log likelihood surfaces computed using the planar and network KDE methods. To recap, these represent the most likely bandwidths that would lead to the distribution of crime events observed examined during the (60-day) calibration window. In both cases, a well-defined maximum is evident; while this is less visually striking in the case of the network KDE, the optimum is readily discernible when the data are rescaled to show only the highest likelihood values. The optimal bandwidths are 55 days and 610 m for the planar KDE and 78 days and 820 m for the network KDE, and these values are used in what follows.

That the spatial bandwidths for the two methods differ is intuitively reasonable for two reasons. First, the network distance between two points will always be greater than or equal to the straight-line distance. Moreover, according to the theory discussed above, network distance better reflects the urban reality and should better encode offender activity/awareness spaces. The difference in temporal bandwidths is not readily explained in terms of simple differences in the physical properties of the network or grid used; however, it should be emphasised that spatial and temporal bandwidths would not necessarily be expected to be independent in this sense. The change in spatial structure may simply mean that the greatest performance is found by including events that occur over longer temporal scales: the two methods simply capture different incidents. Regardless of the reason for the disparity, though, it is worth noting that the network KDE generates substantially higher log likelihood values around the optimum, suggesting that it is has greater predictive power, at least for the validation data.

The apparently rapid drop in likelihood for smaller spatial bandwidths occurs as a result of the estimation procedure used, which is necessary to avoid numerical errors that can occur in the computation of the likelihood values (see “Methods” section). This leads to the visible ‘shelf’ at around 300 m for the planar method and 450 m for the network method. However, this is not an artefact of the procedure: models with shorter bandwidths are simply found to fit the data very poorly.

### Crime Risk Heatmaps

**Fig. 7:**
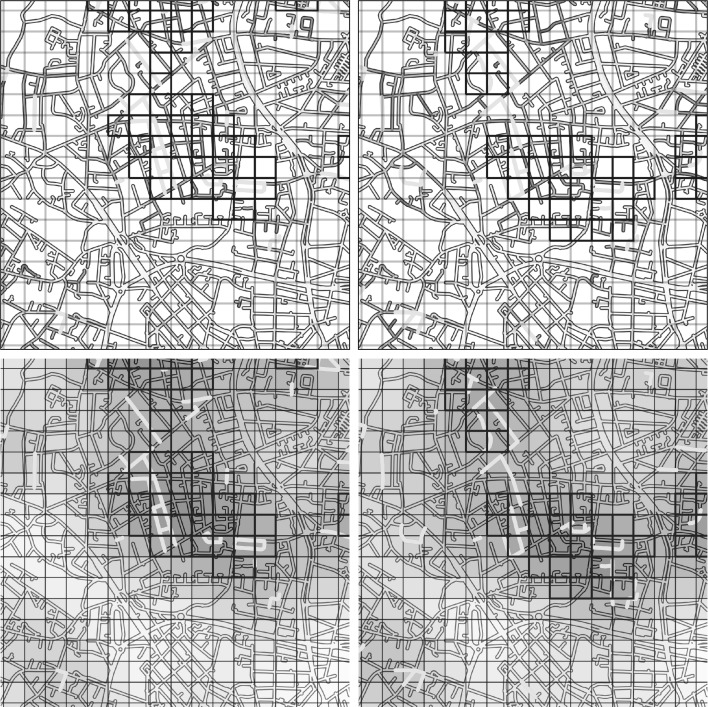
(*Top row*) Network heatmaps showing the predicted risk surface on the 26 February 2014 (*left*) and 27 April 2014 (right). *Darker shades* of *red* indicate higher risk. Grid squares are overlaid for comparison. (*Bottom row*) Grid heatmaps for the same two dates. *Darker shades of red* indicate higher risk. (All figures) Grid *squares* marked in *dark blue* and street segments marked in *light blue* are in the top 1 % ranked by predicted risk (Color figure online)

Having computed optimal bandwidth parameters using the validation data, it is possible to generate predicted risk surfaces for a prospective time window of 1 day. Figure 7 shows surfaces for two different days (2 months apart), computed with the new network- and grid-based KDE methods. Comparing the left and right panels, we see examples of persistent risk, such as the hot areas close to the central and east sides of the plot, and more dynamic shifts in the risk surface, such as the group of roads in the south-west corner. This is to be expected given the theoretical perspectives discussed in the introduction.

Figure 7 illustrates several important differences between the two KDE approaches. First, comparing the top and bottom panels it is clear that the output of the NTKDE is significantly more specific, showing at a glance which street segments in the displayed region have the highest anticipated risk. As discussed previously, multiple segments generally intersect a single grid cell, meaning that targeting a single cell is a challenge for police officers without information about where they should attend within that cell. Several examples are also readily apparent in which the grid-based method fails to identify small hotspots identified by the network-based approach. For example, the risky groups of streets in the northwest, northeast, and southwest corners are not flagged by the grid-based approach for this reason: their risk is effectively ‘diluted’ across multiple grid squares and therefore they are not highly ranked. Conversely, 40–50 % of the top-ranked grid cells do not contain any portions of hot street segments. This occurs because the grid-based approach ignores the underlying street structure within the city, permitting risk to diffuse in all directions regardless of the physical reality. These cells are therefore spurious detections that are flagged only because of their Euclidean proximity to a number of other crimes.

### Predictive Accuracy

**Fig. 8:**
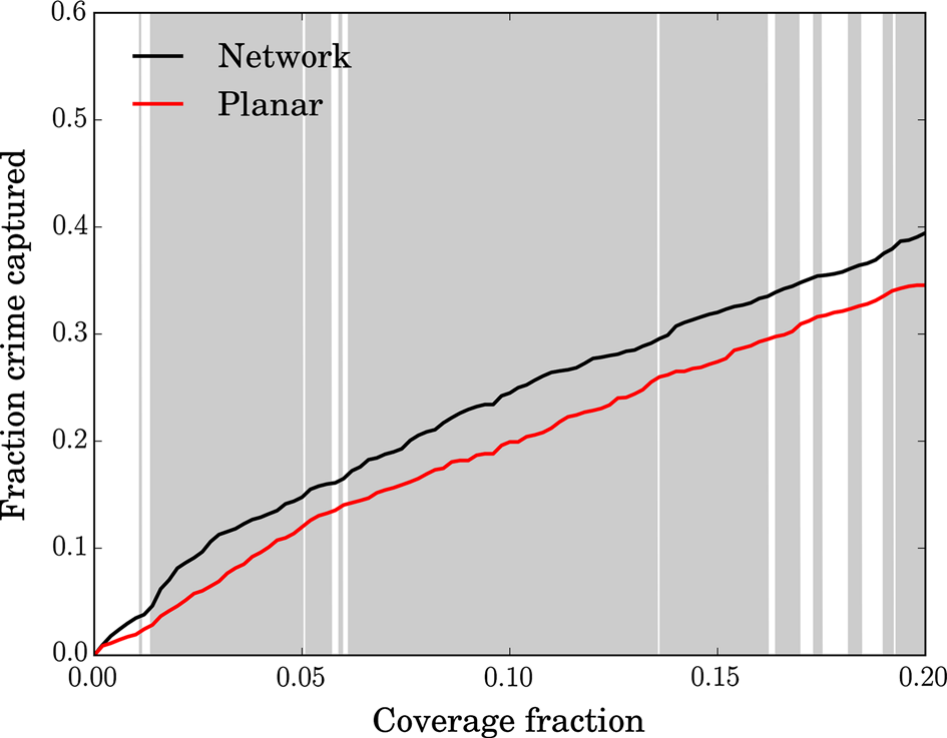
Mean hit rate against coverage for the network and grid-based KDE prediction approaches, computed over 90 consecutive days

Figure 8 shows the mean hit rate, aggregated over the 90 days of testing data, for the NTKDE and STKDE approaches. As discussed (see “Methods” section), the notion of coverage has different meanings in the network and planar contexts. In order to ensure a fair comparison, we therefore evaluate the performance of the STKDE using the ‘network intersection coverage’ method described in “Methods” section. This takes into account the variation in street length between grid cells, since the percentage coverage is based on the total length of road inside the selected squares. This means that grid cells which contain greater road length represent a greater fraction of coverage than would be estimated on the basis of their area alone. Figure 8 shows the overall hit rates of the two approaches when coverage is calculated on this basis. The background shading indicates coverage levels where the NTKDE hit rate is significantly higher than the STKDE approach at the 5 % significance level, assessed using the WSR test. We verified that the difference in daily hit rates for the two methods does not have any significant autocorrelation ($p=0.05$) up to a lag of 21 days, as this is a required assumption of this approach. For this comparison, the NTKDE is more accurate per unit of road considered, having a hit rate that is 1.2 times as good as the grid-based equivalent for most coverage levels.

**Fig. 9:**
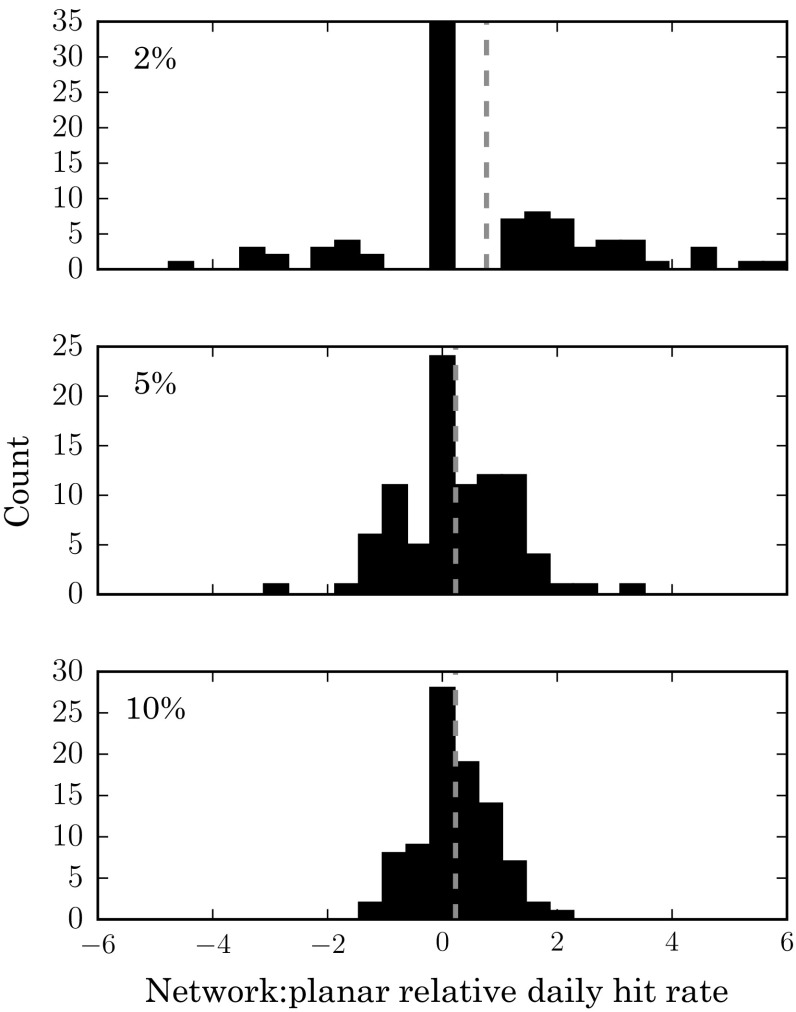
Histograms showing the predictive accuracy of the NTKDE relative to the STKDE at three different coverage levels (2, 5 and 10 %). The improvement factor is given by the difference in daily hit rate between the two methods divided by the mean planar hit rate. Positive values indicate that the NTKDE performed better. The *red dashed line* indicates the mean value in each case

As the above analysis uses data aggregated across all days, it is possible (though unlikely) that the result obtained is influenced by a small number of unusual days where the NTKDE performs substantially better than the STKDE. Thus, to gain greater insight into the relative performance of the two methods, we also assessed the daily difference in hit rate over the 90 days of test data. For this purpose, we define the relative hit rate as the absolute difference in the daily hit rates (for a given coverage, such as 10 % of the study area) between the network and grid-based approaches, divided by the mean grid-based hit rate (measured by network intersection coverage) over the full testing dataset. A positive value therefore indicates that the network method was more effective on a given day. A value of 0 represents identical performance from the two methods, for example, while a value of 1 indicates that the network method was approximately twice as accurate as the grid-based approach. The results are shown in Fig. 9 for three different coverage levels (2, 5 and 10 %). At a coverage level of 2 %, the two methods have equivalent accuracy on approximately one third of days. However, on around one quarter of the 90 days the NTKDE outperforms the STKDE by a factor of 2 or more. At a coverage level of 10 %, the distribution is narrower, but on over a quarter of the days the difference in performance is over half of the mean STKDE performance in favour of the NTKDE. Overall, the mean of these values at a coverage level of 10 % is 0.25, which corresponds to a mean improvement of 25 % on a daily basis. The WSR test confirms that the results are highly statistically significant, with the NTKDE performing better at the majority of coverage levels between 1 and 20 %.

## Discussion

In the last decade, research on the spatial (and temporal) distribution of crime has begun to move from the task of description to that of prediction. To date, for almost all attempts at prediction, the units of analysis considered have either been areas (e.g. police beats), or regular (often arbitrarily) sized grid cells. This, however, fails to account for the effect of a key element of the urban backcloth, upon which much human activity takes place: the street network. Such activity includes the movement of ordinary citizens through places, which influences the conditions for crime and the activities of offenders and the police. With this in mind, in this paper we have introduced a network-based method for the prospective identification of crime locations. The aim of our work was to build on existing predictive approaches, while taking advantage of the theoretical and practical advantages of the network setting.

Our technical contributions are two-fold: we have shown how spatio-temporal kernel methods can be translated into network space, and have introduced a fitting procedure which allows the optimal parameters for such a model to be identified. Furthermore, we have shown how the accuracy of network-based predictions can be measured in such a way that they can meaningfully be compared with grid-based alternatives. When applied to a sample of residential burglary data for a major city in the UK, the performance of the algorithm—tested over multiple (90) days—is found to be substantially better than that of its grid-based counterpart, with, for example, 20 % more crime identified at a coverage level of 5 %. Statistical analysis confirms that the improvement in accuracy is highly significant at coverages in the range 1–20 %. We now consider the theoretical and practical interpretation of these results.

The basis for our predictive approach is, ultimately, the empirically-observed tendency of crime to concentrate in space: it is this fact that suggests that a disproportionate volume of crime can be captured by identifying a small fraction of places. It is clear, therefore, that Weisburd (2015) ‘law of crime concentration’ is highly relevant to our work. Most immediately, it suggests that concentration of this type is ubiquitous in real-world, and that—crucially for our approach—it is meaningfully manifested at the street segment level. This finding alone represents a strong rationale for the development of network-based prediction methods. In addition, however, the law’s prediction that levels of concentration are consistent across contexts also has implications for work in this area. This suggests that the volume of crime that can potentially be captured at small spatial scales—essentially the ‘predictability’ of crime—will be broadly similar across settings. This defines a clear objective for prediction: if it can be said with confidence that *Y*% of incidents will be located on *X*% of street segments, then the task of an algorithm is to identify those segments.

In fact, a dynamic predictive algorithm such as ours seeks to go further than this, by exploiting day-to-day fluctuations in the distribution of crime. Even while accounting for the law of crime concentration, significant variation is likely to be seen in the precise daily distribution of crime: the most risky streets over a period of 1 year (for example) will not necessarily be the most risky on every individual day within it. Indeed, research on space-time clustering suggests that this will not be the case, and that significant short-term effects can be seen in the spatial distribution of crime. Dynamic models such as ours seek to capitalise on this: rather than identifying the *X*% highest-risk streets across an extended period, a different *X*% of segments can be chosen each day. Considered in this way, the law of crime concentration effectively represents a lower limit on the potential ‘hit rate’ of a dynamic model: if segments can be re-ordered each day, then the level of concentration is guaranteed to be at least as pronounced as it is when aggregated over a longer period.

From the perspective of the law of crime concentration, our results have several implications. Most immediately, they underline the significance of the street segment as a meaningful and useful unit of analysis in understanding the spatial distribution of crime. Our findings show that not only do segment-level effects account for a large amount of risk variation (as demonstrated previously; see Steenbeek and Weisburd 2016), but that this variation translates into predictive value. In addition to this, the fact that our model is dynamic has a number of consequences: if its relative success can be taken as evidence that it consistently identifies the locations of greatest crime concentration, then this implies that exactly which set of micro-places account for the greatest risk changes somewhat over time (for residential burglary at least). This, it should be noted, is perfectly consistent with the law of crime concentration: the fact that levels of concentration are stable over time does not mean that the actual locations of crime are. Rather, it simply highlights the importance of considering both the spatial and temporal dimensions when examining crime concentration. Indeed, examining the level of overlap between time periods in terms of the locations of greatest crime concentration may be an interesting topic for future research.

Further research is also necessary to explore other ways in which the algorithm can be optimised, both in technical and practical terms. One particular issue concerns the temporal unit of analysis for which predictions are generated. In this work, we focused on a prediction window of 1 day. While convenient, this may fail to capture some effects since it has been shown that crime levels vary by time of day (Felson and Poulsen 2003), and a number of studies have demonstrated the utility of examining risk over the course of the day (Ratcliffe 2004; Johnson et al. 2007b; Sagovsky and Johnson 2007). There is clearly a middle ground to be found with respect to the frequency with which maps are generated: it may be advantageous to capture time-of-day effects, but at present very frequent updating is likely to be excessive and may be difficult to act upon operationally. The utility of real-time updating should be explored in future research both in terms of practical implications and algorithmic accuracy. Exploration of practical deployment issues will require a different approach to that taken here, and will likely involve field research to establish the various constraints within which the police operate. In terms of technical issues, our approach can, in theory, be applied at any temporal scale with no modifications required, but two practical concerns exist. The first is that for some offences the exact time a crime occurs will be unknown. For example, in the case of burglary, offences generally occur when the victim is away from home and so only the interval over which an offence could have occurred will be known with certainty. For burglary, this will typically be up to an eight-hour (or so) time window (Ratcliffe 2004; Sagovsky and Johnson 2007) meaning that it may not be possible to reliably establish the accuracy of predictions produced for shorter intervals. The second issue is that reducing the time aggregation window will result in fewer crimes per prediction, which we expect to produce greater variation around the mean hit rate. This too may make the estimation of the accuracy of methods less reliable. For now, we suggest that future work should explore the accuracy of algorithms over ‘periods’ or ‘shifts’ of the day that (ideally) mirror those used for police resourcing.

A number of further theoretical implications also arise from our findings. In motivating the model presented, we have built upon existing theory concerned with offender targeting strategies in general, and near repeat victimisation in particular. Specifically, we have elaborated upon the offender-as-forager hypothesis, which itself builds upon crime pattern theory and ideas from behavioural ecology. In doing so, we have considered more thoroughly how the street network might shape the dynamic development of offender awareness of criminal opportunities (see also Johnson 2014), and how this in turn might lead to the propagation of risk though the street network. With regard to the relationship between network structure and criminal behaviour, our results can thus be considered to provide further support for the offender-as-forager hypothesis and crime pattern theory. Further work, however, will be required to establish precisely how the propagation of risk occurs, what factors (if any) influence it, and whether it can be encoded in a more nuanced way than was used here. To elaborate, in the current model, the anticipated risk from each crime propagates through the network from one street segment to the next, decaying in intensity as network distance increases. However, factors other than the structure of the street network may influence whether risk spreads from one street segment to another. For example, risk may be more likely to spread to street segments that contain similar homes, that are perhaps near to other (criminogenic) facilities (e.g. Bernasco and Block 2011), or to those street segments that are located within areas with low social cohesion (e.g. Sampson et al. 1997). Examining such issues represents an agenda for future research. Here we simply wish to assert promise for approaches that consider theoretical reasons for risk propagation. They could usefully be compared in future studies with scanning approaches based on networks, such as that offered by Shiode and Shiode (2014).

Our algorithm, and general approach, have potential to be of practical use in real-world policing contexts. While the predictive accuracy alone suggests that the method would compare favourably with other existing approaches, there are a number of logistical reasons why it may also be beneficial. Predictions expressed in terms of specific street segments, for example, are particularly well-suited to operational deployment, since it is in exactly these terms that police officers would typically plan their routes. This may be of benefit with respect to implementation compliance, which is known to be a critical issue in the context of targeted policing initiatives. In general, such predictions would appear to offer a number of advantages: they offer an unambiguous indication of where officers should patrol which, in turn, is verifiable by senior officers. A further potential advantage is that police presence on road networks does not need to be restricted to on-foot officers. These maps offer more usability to vehicular patrols, and could potentially lead to the development of intelligent routing procedures that enable visible presence through police cars on route to non-urgent calls. Generally, joining up the segments of high risk into the most convenient (e.g. shortest or least fragmented) patrol routes appears to be a means of making such maps as usable as possible.

This point does highlight the fact that, despite their promise, there are further questions to be answered concerning the practical implications of street network based prediction approaches. In the original description of the prospective mapping approach, Bowers et al. (2004) discussed the extent to which resulting areas of predicted risk were themselves clustered or, in contrast, more dispersed. This affects the practicality of resulting maps for patrolling—if identified segments are dispersed, a lot of time may be spent travelling between them—and has further implications for the law of concentration at places. Further research might compare predictive techniques on dispersal metrics to discern whether network approaches lead to more fragmentation. Such metrics could also examine typical patterns of fragmentation of high risk segments more generally, to add to the crime concentration literature. In any case, we would argue that due to their nature, segments are inherently more ‘connectable’ spatially than their grid based counterparts.

There are a number of other potential avenues for further research in this domain. Most obviously, there is a need to test the performance of the algorithms in alternative settings: in other cities and countries, and for other crime types. In addition to this, however, there is broad scope for the development of algorithmic variations. In this work, we chose a relatively simple predictive algorithm—a kernel-based approach—in order to place the emphasis on the network context (and to avoid confounding the comparisons made). The general framework, however, could be applied to any predictive method, including others which have performed well in grid-based implementations. The adaptation of these algorithms, and the development of novel approaches tailored to the network context in particular, represents an exciting direction for further work.

## References

[CR1] Andresen MA, Malleson N (2011). Testing the stability of crime patterns: implications for theory and policy. J Res Crime Delinq.

[CR2] Arlot S, Celisse A (2010). A survey of cross-validation procedures for model selection. Stat Surv.

[CR3] Armitage R, Farrell G, Bowers KJ, Johnson SD, Townsley M (2007). Sustainability versus safety: confusion, conflict and contradiction in designing out crime. Imagination for crime prevention: essays in honour of Ken Pease vol. 21 of crime prevention studies.

[CR4] Ashby MP, Bowers KJ (2013). A comparison of methods for temporal analysis of aoristic crime. Crime Sci.

[CR5] Ashton J, Brown I, Senior B, Pease K (1998). Repeat victimisation: offender accounts. Int J Risk Secur Crime Prev.

[CR6] Beavon DJK, Brantingham PL, Brantingham PJ, Clarke RV (1994). The influence of street networks on the patterning of property offences. Crime Prev Stud.

[CR7] Bennett T, Wright R (1984). Burglars on burglary: prevention and the offender.

[CR8] Bergmeir C, Benítez JM (2012). On the use of cross-validation for time series predictor evaluation. Inf Sci.

[CR9] Bernasco W (2008). Them again? Same-offender involvement in repeat and near repeat burglaries. Eur J Criminol.

[CR10] Bernasco W, Block R (2011). Robberies in Chicago: a block-level analysis of the influence of crime generators, crime attractors, and offender anchor points. J Res Crime Delinq.

[CR11] Bernasco W, Johnson SD, Ruiter S (2015). Learning where to offend: effects of past on future burglary locations. Appl Geogr.

[CR12] Bollobás B (2002). Modern graph theory.

[CR13] Bowers K (2014). Risky facilities: crime radiators or crime absorbers? A comparison of internal and external levels of theft. J Quant Criminol.

[CR14] Bowers KJ, Johnson SD, Gill M (2014). Crime mapping as a tool for security and crime prevention. The handbook of security.

[CR15] Bowers KJ, Johnson SD, Guerette RT, Summers L, Poynton S (2011). Spatial displacement and diffusion of benefits among geographically focused policing initiatives: a meta-analytical review. J Exp Criminol.

[CR16] Bowers KJ, Johnson SD, Pease K (2004). Prospective hot-spotting: the future of crime mapping?. Br J Criminol.

[CR17] Braga AA, Hureau DM, Papachristos AV (2011). The relevance of micro places to citywide robbery trends: a longitudinal analysis of robbery incidents at street corners and block faces in Boston. J Res Crime Delinq.

[CR18] Braga AA, Papachristos AV, Hureau DM (2014). The effects of hot spots policing on crime: an updated systematic review and meta-analysis. Justice Q.

[CR19] Brantingham PL, Brantingham PJ (1993). Environment, routine and situation: toward a pattern theory of crime. Adv Criminol Theory.

[CR20] Brantingham PL, Brantingham PJ, Vajihollahi M, Wuschke K, Weisburd D, Bernasco W, Bruinsma GJ (2009). Crime analysis at multiple scales of aggregation: a topological approach. Putting crime in its place.

[CR21] Budd T (2001). Practice Messages from the Britsh Crime Survey. No. 5/01 in Home Office Statistical Bulletin.

[CR22] Cheng T, Adepeju M (2013) Detecting emerging space-time crime patterns by prospective STSS. In: Proceedings of the 12th international conference on geocomputation

[CR23] Clarke RV, Cornish DB (1985). Modeling offenders’ decisions: a framework for research and policy. Crime Justice.

[CR24] Clarke RV, Eck JE (2005). Crime analysis for problem solvers.

[CR25] Cohen LE, Felson M (1979). Social change and crime rate trends: a routine activity approach. Am Sociol Rev.

[CR26] Davies T, Johnson SD (2014). Examining the relationship between road structure and burglary risk via quantitative network analysis. J Quant Criminol.

[CR27] Davies TP, Bishop SR (2013). Modelling patterns of burglary on street networks. Crime Sci.

[CR28] Diebold FX, Mariano RS (1995). Comparing predictive accuracy. J Bus Econ Stat.

[CR29] Everson S, Pease K, Farrell G, Pease K (2001). Crime against the same person and place: detection opportunity and offender targeting. Repeat victimization, vol. 2 of crime prevention studies.

[CR30] Farrell G, Phillips C, Pease K (1995). Like taking candy: Why does repeat victimization occur?. Br J Criminol.

[CR31] Felson M, Poulsen E (2003). Simple indicators of crime by time of day. Int J Forecast.

[CR32] Fielding M, Jones V (2012). ‘Disrupting the optimal forager’: predictive risk mapping and domestic burglary reduction in Trafford, Greater Manchester. Int J Police Sci Manag.

[CR33] Forrester D, Chatterton M, Pease K, Brown R (1988). The Kirkholt burglary prevention project, Rochdale.

[CR34] Genton MG (2002). Classes of kernels for machine learning: a statistics perspective. J Mach Learn Res.

[CR35] Goldstein H (1990). Excellence in problem-oriented policing.

[CR36] Gorr W, Olligschlaeger A (2002). Crime hot spot forecasting: modeling and comparative evaluation.

[CR37] Groff ER, La Vigne NG, Tilley N (2002). Forecasting the future of predictive crime mapping. Analysis for crime prevention, vol. 13 of crime prevention studies.

[CR38] Grove LE, Farrell G, Farrington DP, Johnson SD (2012). Preventing repeat victimization: a systematic review.

[CR39] Hastie T, Tibshirani R, Friedman J (2009). The elements of statistical learning: data mining, inference, and prediction. Springer series in statistics.

[CR40] Jiang B, Claramunt C (2004). Topological analysis of urban street networks. Environ Plan B Plan Des.

[CR41] Johnson SD (2008). Repeat burglary victimisation: a tale of two theories. J Exp Criminol.

[CR42] Johnson SD (2014). How do offenders choose where to offend? Perspectives from animal foraging. Leg Criminol Psychol.

[CR43] Johnson SD, Bernasco W, Bowers KJ, Elffers H, Ratcliffe J, Rengert G, Townsley M (2007). Space-time patterns of risk: a cross national assessment of residential burglary victimization. J Quant Criminol.

[CR44] Johnson SD, Birks DJ, McLaughlin L, Bowers KJ, Pease K (2007b). Prospective mapping in operational context: Home Office Online Report 19/07. Home Office, London

[CR45] Johnson SD, Bowers KJ (2004). The burglary as clue to the future: the beginnings of prospective hot-spotting. Eur J Criminol.

[CR46] Johnson SD, Bowers KJ, Farrell G, Bowers KJ, Johnson SD, Townsley M (2007). Burglary prediction: theory, flow and friction. Imagination for Crime Prevention: essays in honour of Ken Pease , vol. 21 of crime prevention studies.

[CR47] Johnson SD, Bowers KJ (2010). Permeability and burglary risk: Are Cul-de-Sacs safer?. J Quant Criminol.

[CR48] Johnson SD, Bowers KJ, Bruinsma G, Weisburd D (2014). Near repeats and crime forecasting. Encyclopedia of criminology and criminal justice.

[CR49] Johnson SD, Bowers KJ, Birks DJ, Pease K, Weisburd D, Bernasco W, Bruinsma GJ (2009). Predictive mapping of crime by promap: accuracy, units of analysis, and the environmental backcloth. Putting crime in its place.

[CR50] Johnson SD, Summers L, Pease K (2009). Offender as Forager? A direct test of the boost account of victimization. J Quant Criminol.

[CR51] Kennedy LW, Caplan JM, Piza E (2011). Risk clusters, hotspots, and spatial intelligence: risk terrain modeling as an algorithm for police resource allocation strategies. J Quant Criminol.

[CR52] Knutsson J, Clarke RV (2006). Putting theory to work: implementing situational prevention and problem-oriented policing , vol. 20 of crime prevention studies.

[CR53] Lasley J (1998). Using traffic barriers to design out crime: a program evaluation of LAPD’s Operation Cul de Sac.

[CR54] Mohler GO, Short MB, Brantingham PJ, Schoenberg FP, Tita GE (2011). Self-exciting point process modeling of crime. J Am Stat Assoc.

[CR55] Morgan F, Farrell G, Pease K (2001). Repeat burglary in a Perth suburb: indicator of short-term or long-term risk?. Repeat victimisation, vol. 12 of crime prevention studies.

[CR56] Okabe A, Satoh T, Sugihara K (2009). A kernel density estimation method for networks, its computational method and a GIS-based tool. Int J Geogr Inf Sci.

[CR57] Olligschlaeger A, Weisburd D, McEwen T (1997). Artificial neural networks and crime. Crime mapping and crime prevention, vol. 8 of crime prevention studies.

[CR58] Osborn DR, Tseloni A (1998). The distribution of household property crimes. J Quant Criminol.

[CR59] Pease K (1998). Repeat victimisation: taking stock.

[CR60] Pitcher AB, Johnson SD (2011). Exploring theories of victimization using a mathematical model of burglary. J Res Crime Delinq.

[CR61] Polvi N, Looman T, Humphries C, Pease K (1991). The time course of repeat burglary victimization. Br J Criminol.

[CR62] Porta S, Crucitti P, Latora V (2006). The network analysis of urban streets: a dual approach. Phys A Stat Mech Appl.

[CR63] Porta S, Crucitti P, Latora V (2006). The network analysis of urban streets: a primal approach. Environ Plan B Plan Des.

[CR64] Ratcliffe JH (2004). The hotspot matrix: a framework for the spatio-temporal targeting of crime reduction. Police Prac Res.

[CR65] Rey SJ, Mack EA, Koschinsky J (2012). Exploratory space-time analysis of burglary patterns. J Quant Criminol.

[CR66] Robinson W (1950). Ecological correlations and the behavior of individuals. Am Sociol Rev.

[CR67] Sagovsky A, Johnson SD (2007). When does repeat burglary victimisation occur?. Aust N Z J Criminol.

[CR68] Sampson RJ, Raudenbush SW, Earls F (1997). Neighborhoods and violent crime: a multilevel study of collective efficacy. Science.

[CR69] Sheather SJ (2004). Density estimation. Stat Sci.

[CR70] Sherman LW, Gartin PR, Buerger ME (1989). Hot spots of predatory crime: routine activities and the criminology of place. Criminology.

[CR71] Shiode S, Shiode N (2014). Microscale prediction of near-future crime concentrations with street-level geosurveillance. Geogr Anal.

[CR72] Short MB, D’Orsogna MR, Pasour VB, Tita GE, Brantingham PJ, Bertozzi AL, Chayes LB (2008). A statistical model of criminal behavior. Math Models Methods Appl Sci.

[CR73] Steenbeek W, Weisburd D (2016). Where the action is in crime? An examination of variability of crime across different sapatial units in The Hague, 2001–2009. J Quant Criminol.

[CR74] Summers L, Johnson SD, Rengert GF, Bernasco W (2010). The use of maps in offender interviewing. Offenders on offending: learning about crime from criminals.

[CR75] Summers L, Johnson SD (2016) Does the configuration of the street network influence where outdoor serious violence takes place? Using space syntax to test crime pattern theory. J Quant Criminol, 1–24

[CR76] Townsley M, Homel R, Chaseling J (2000). Repeat burglary victimisation: spatial and temporal patterns. Aust N Z J Criminol.

[CR77] Townsley M, Homel R, Chaseling J (2003). Infectious burglaries: a test of the near repeat hypothesis. Br J Criminol.

[CR78] Townsley M, Sidebottom A (2010). All offenders are equal, but some are more equal than others: variation in journeys to crime between offenders. Criminology.

[CR79] Weisburd D (2015). The law of crime concentration and the criminology of place*. Criminology.

[CR80] Weisburd D, Groff ER, Yang S-M (2012). The criminology of place.

[CR81] Wiles P, Costello A (2000) The ‘road to nowhere’: The evidence for travelling criminals. No. 207 in Home Office Research Studies. Home Office Research, Development and Statistics Directorate, London

